# Epidemic Process over the Commute Network in a Metropolitan Area

**DOI:** 10.1371/journal.pone.0098518

**Published:** 2014-06-06

**Authors:** Kenta Yashima, Akira Sasaki

**Affiliations:** 1 Department of Evolutionary Studies of Biosystems (Sokendai-Hayama), The Graduate University for Advanced Studies (Sokendai), Hayama, Kanagawa, Japan; 2 Evolution and Ecology Program, International Institute for Applied Systems Analysis, Laxenburg, Austria; 3 Precursory Research for Embryonic Science and Technology (PRESTO), Japan Science and Technology Agency (JST), Kawaguchi, Saitama, Japan; 4 Meiji Institute for Advanced Study of Mathematical Sciences, Meiji University, Nakano, Tokyo, Japan; Argonne National Laboratory, United States of America

## Abstract

An understanding of epidemiological dynamics is important for prevention and control of epidemic outbreaks. However, previous studies tend to focus only on specific areas, indicating that application to another area or intervention strategy requires a similar time-consuming simulation. Here, we study the epidemic dynamics of the disease-spread over a commute network, using the Tokyo metropolitan area as an example, in an attempt to elucidate the general properties of epidemic spread over a commute network that could be used for a prediction in any metropolitan area. The model is formulated on the basis of a metapopulation network in which local populations are interconnected by actual commuter flows in the Tokyo metropolitan area and the spread of infection is simulated by an individual-based model. We find that the probability of a global epidemic as well as the final epidemic sizes in both global and local populations, the timing of the epidemic peak, and the time at which the epidemic reaches a local population are mainly determined by the joint distribution of the local population sizes connected by the commuter flows, but are insensitive to geographical or topological structure of the network. Moreover, there is a strong relation between the population size and the time that the epidemic reaches this local population and we are able to determine the reason for this relation as well as its dependence on the commute network structure and epidemic parameters. This study shows that the model based on the connection between the population size classes is sufficient to predict both global and local epidemic dynamics in metropolitan area. Moreover, the clear relation of the time taken by the epidemic to reach each local population can be used as a novel measure for intervention; this enables efficient intervention strategies in each local population prior to the actual arrival.

## Introduction

A theoretical understanding of the epidemic spread of an infectious disease within a metropolitan area is essential for its prevention and control. In this study, we analyze the epidemic dynamics of the spread of an infectious disease, such as influenza, over the commute network in a metropolitan area. We particularly focus on such theoretical aspects as how the epidemiological parameters and statistical properties of the commute network affect the probability that an infectious disease invades and spreads out globally throughout the area, the final size of the global epidemic, and the time until the epidemic attains its peak, as these aspects would provide valuable insights into the prevention and control of a disease within a metropolitan area. For this purpose, we simulated the spread of infection over the commute network in the Tokyo metropolitan area using an individual-based model (IBM) in which each individual's daily commute movements are simulated on the basis of the actual commute data for the Tokyo metropolitan area. We then constructed a simple, analytically tractable mathematical model that could reproduce the behavior of epidemics on the commute network observed in the IBM.

Previous simulation studies of epidemic dynamics on human networks have been effective for assessing various intervention strategies, such as quarantine, vaccination, and antiviral drug treatments. Strategies for containing the emerging influenza in Southeast Asia were evaluated by Longini et al. [1] and Ferguson et al. [2]. Other studies have measured the effects of mitigation strategies on influenza pandemics within Great Britain [3] and the United States of America [3], [4]. These analyses were based on spatially explicit disease transmission models in which the details of the population structure, such as the age structure and locations within the areas (e.g., households, schools, workplaces, and shops) were taken into account; moreover, the daily commute movements and the travels of each individual were considered. There have also been a few theoretical studies of pandemic influenza in the Tokyo metropolitan area in which daily commute movements were considered. Ohkusa and Sugawara [5], [6] utilized Person-Trip data to simulate individual movements and constructed a detailed simulation model in order to evaluate the effects of a quarantine policy. Yasuda et al. [7], [8] and Saito et al. [9] performed a similar analysis based on actual demographic data by constructing a suburban community along a commuter line. These studies aimed to analyze the outcomes of various intervention strategies in each specific scenario and evaluate their efficacies quantitatively to form a basis for policy-making. However, as these analyses focused only on specific cases, they did not examine how various aspects of the commute network affect the epidemic dynamics. In other words, these analyses were restricted only to particular areas and cities and could not provide a general understanding of different locations. Therefore, a more general understanding of the effects of the geographic and social structures of the population on the epidemic dynamics is needed.

When the contact between individuals shows geographical and social localization, a theory of networks can be used to analyze the spread of infectious diseases. Contact network models, in which each node represents an individual, have been used to analyze various aspects of epidemic dynamics and revealed the relation between the network characteristics and the properties of the epidemic process (e.g., the epidemic thresholds for various parameters, final epidemic size, and immunization threshold) [10][Chapter 9 and references therein]. In contrast, when populations consist of well-mixed subpopulations (e.g., urban communities, geographical regions, and social groups), metapopulation network models are more suitable as a theoretical framework. In such models, individuals inhabit each node (i.e., subpopulation), and their movements between the nodes couple the subpopulations to each other. For the case in which the mobility patterns represent random movement of individuals, Colizza and Vespignani [11], [12] have shown the existence of a global epidemic threshold and derived an explicit analytic expression that is analogous to the basic reproductive ratio. They also showed that the mobility of the individuals is crucial to the epidemic dynamics and that heterogeneity promotes global epidemics. Balcan and Vespignani [13], [14] further extended these studies to the case in which the mobility patterns are recurrent, such as commuting (i.e., individuals return to their original locations). They derived a similar formula for the global epidemic threshold, which showed that the rate of commuting from the original location and the amount of time spent at the destination are critical determinants for disease invasion. The importance of the individual's recurrent mobility patterns to the epidemic dynamics was also addressed recently by Keeling et al. [15] and Belik et al. [16]. The theoretical framework of the metapopulation network has thus enabled us to analytically deal with the epidemic process in structured populations and revealed the relation between the commute network structures and the epidemic dynamics. However, most studies have adopted a randomly generated complex network with a given degree distribution, ignoring, for example, the correlated connections between subpopulations as well as the recurrent mobility, both of which are often substantial in actual commute networks (however, refer Keeling et al. [15], Belik et al. [16] and Eubank et al. [17]).

In the present study, we analyze the epidemic process over the commute network in the Tokyo metropolitan area. For this purpose, we developed an individual-based model for the epidemic process over the actual commute network in the Tokyo metropolitan area. We formulated the model as a metapopulation network model in which each commuter train station is associated with a residential area (home population) and a business area (work population). As actual commuting data are utilized, this metapopulation network inherently includes the relevant geographic and social structures. We used the susceptible, infectious, recovered/removed model [18] to describe the disease transmission and actual commute data from the Tokyo metropolitan area to simulate the individuals' movements. To determine the aforementioned theoretical aspects, we calculated the probability of a global epidemic, the final size of the global epidemic, the time until the global epidemic attain its peak, and the final size and arrival time of the epidemic in each local population, all under various epidemic parameters. We then used these values to assess the conditions under which a global epidemic would occur and evaluate the extent and severity of the resulting damage. The epidemic parameters we considered include the location at which the disease invasion originated and the local population size thereof, which have not been emphasized in previous studies. With this focus, we find that for a fixed set of disease parameters, the probability of a global epidemic is determined by the local population size of the original location of the infection, whereas the geographical location of the site of origin within the commute network has little effect on the outcome. Furthermore, we compared the results of a randomly reconnected network model, which only inherited the connection probability between the subpopulations' size classes from the actual network, to examine the extent to which detailed information regarding network connections, such as the geographical locations of subpopulations and the specific connectivity between them, is necessary. We performed extensive Monte Carlo simulations in an attempt to derive a general relation that would enable the prediction of epidemic dynamics. We found, for example, that there is a simple relation between the size of a local population and the time until an epidemic reaches it. This knowledge could be used to design a more effective control strategy against infectious disease in a metropolitan area. Finally, we validated these findings using a branching process for the initial spread of infected individuals over the network and using simple difference equations for the epidemiological dynamics over subpopulations with a given size class distribution.

## Methods

### Commute network data for the Tokyo metropolitan area

**Figure 1:**
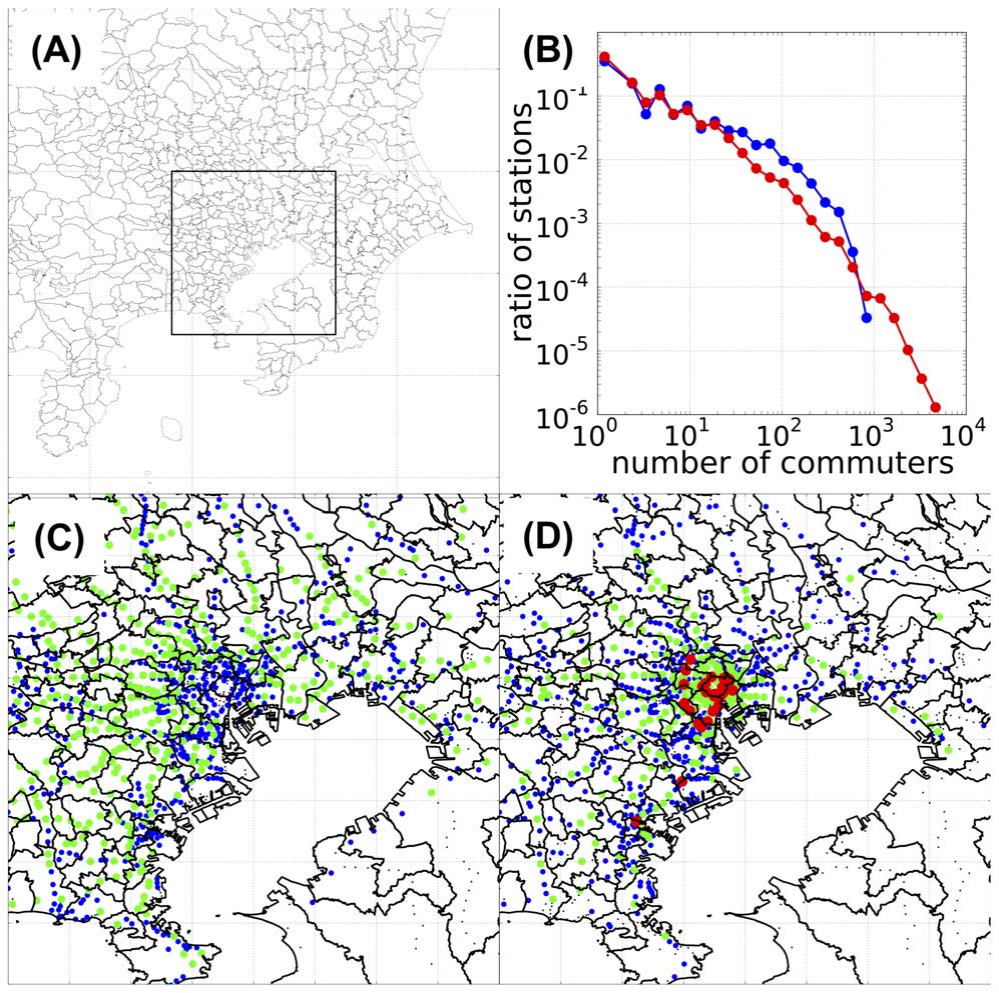
Commuter flow data for the Tokyo metropolitan area. (A) Geographical location of the Tokyo metropolitan area within Kanto region, Japan. The framed rectangle shows the central part of the Tokyo metropolitan area. (B) Distribution of the station sizes on a double-logarithmic plot. Blue line, distribution of home-node stations; red line, distribution of work-node stations. (C) and (D) Geographical distributions of the sizes of home- and work-node stations, respectively, within the central part of the Tokyo metropolitan area. The color indicates the size of the station: black, 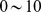 commuters; blue, 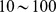 commuters; green, 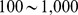 commuters; red, 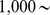 commuters. All numbers are from the 139,841 collected questionnaires of UTC. The red-colored stations in the middle of (D) correspond to Tokyo's inner urban area (along the loop of the Yamanote line); the 2 red stations in the lower left of (D) are the Kawasaki and Yokohama stations. The longitude and latitude of each station were acquired from the Station Database [http://www.ekidata.jp].

The data on commuter flow within the Tokyo metropolitan area were obtained from the Urban Transportation Census (UTC) [19], a survey conducted by the Japanese Ministry of Land, Infrastructure, Transport and Tourism that has been carried out every 5 years since 1960 at three major metropolitan areas of Japan, which are Tokyo, Nagoya, and Osaka regions. The UTC is intended to provide basic data, which is used for preparing public transportation policies in these metropolitan areas. The data contains the results of a questionnaire answered by the users of commuter trains, buses, and streetcars that include the traffic volumes between stations, traffic volumes between bus terminals, and the transportation capacities of public transportation. For information about the commute network in the Tokyo metropolitan area, we used the data from the 10th UTC [19], which was performed in 2005 and is the most recent UTC for which results are available. The region surveyed has a population of approximately 35.6 million and extends to 8 prefectures, which are Tokyo, Kanagawa, Saitama, Chiba, Ibaraki, Gunma, Tochigi, and Yamanashi (Figure 1A). Therefore, the commute data from the UTC covers the entire Tokyo metropolitan area.

The commuter train is the major intra-regional transportation in the Tokyo metropolitan area. According to the UTC data, approximately 40 million people in total use the commuter train daily [19]. The commuter buses are used mainly to access the train stations from residences, workplaces, and schools. Accordingly, the commuter trains are the main determinant of the intra-regional commuting flow within the Tokyo metropolitan area. From the UTC questionnaire survey of commuter train users, we obtained commute data for 139,841 individuals. The data contained information about the commute movements of each individual on a specific day of the survey (November 16–17, 2005). In other words, the train commutes of individuals from their residences to their workplaces or schools on a certain day could be traced.

Each station is specified by a station code. The original UTC data for the Tokyo metropolitan area comprises information for a total of 1,899 stations. The UTC data contains some duplication of station codes, as a single station was assigned a different code for each line. As we are interested in the station nearest the commuter's residence and workplace/school, we used the Station Database [http://www.ekidata.jp] to remove this duplication of station codes, resulting in a total of 1,435 stations specified by newly designated unique station codes. Each of these station codes corresponded to 1 or more stations with a common area of residence and workplace/school. Hereafter, the term “station” is used as a synonym for this newly designated station code. Using these station codes, each individual's commuting pattern can be described by the station of his or her residence (hereafter called the “home population”) and the station of his or her workplace/school (hereafter called the “work population”).

According to the UTC commute data, the traffic volumes of both the home and work populations varied in a few orders of magnitude (Figure 1B). When plotted on a double logarithmic scale, the commuter size distributions of both the home and work populations had slopes of approximately 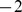 to 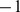. The largest of these commuting populations were present in small but non-negligible numbers. Such long-tailed distributions of commuter sizes have been reported previously [20], and the resulting heterogeneity in the commuting network structure is known to affect epidemic dynamics, especially by lowering the epidemic invasion threshold [11]–[14]. For the home populations, the upper limit of the number of commuters was below 1,000 (this was the number among the total of 139,841 questionnaires collected, which represents only 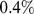 of the total number of residents of the Tokyo metropolitan area; therefore, all figures should be multiplied by a factor of approximately 100 [as not all people are commuting on any given day] when considering the actual population in the Tokyo metropolitan area). On the other hand, the largest work populations exceeded 5,000 commuters (5,000
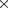
100 in actual numbers), signifying a greater degree of concentration in business areas. The geographical distributions of the population sizes (Figures 1C–D) showed that the larger work populations were concentrated in the inner urban areas, whereas the home populations were distributed over broader areas.

### Individual-based model: Commuting

**Figure 2:**
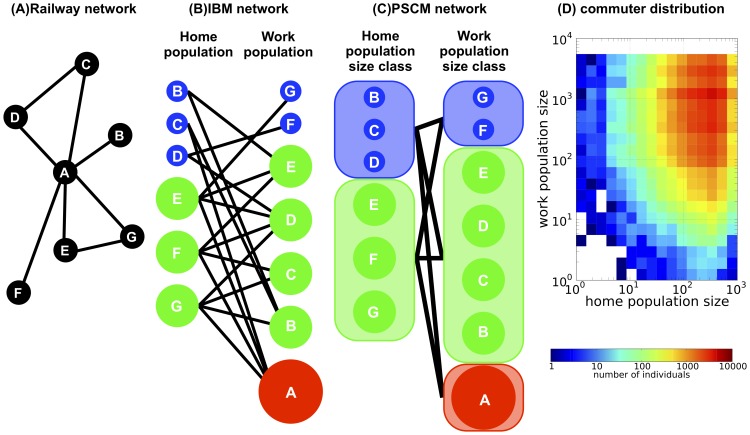
Schematic representation of the population size class model (PSCM). (A) Geographical distribution of the railway network; each node corresponds to a station and each line corresponds to a commuter railway of commuter trains. Every station has a home population (those who reside in the area) and a work population (those who travel to as a workplace/school in the area). There are multiple commuters using the commuter train between each pair of populations. (B) The commuter network utilized in the individual-based model (IBM) calculations. The nodes correspond to the home and work populations of each station, forming a bipartite network in which each line denotes a connection via commuter flow between home and work populations. Local populations with different population sizes are represented by different colors and sizes. (C) The commuter network utilized in the PSCM calculation. Local populations with similar population sizes are grouped into population size classes, which form the nodes, while the total commuter flows between pairs of population size classes form the lines. (D) Joint distribution of the home and work population sizes of commuters in the Tokyo metropolitan area. The number 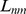 of commuters that live in a home population of size class 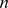 and commute to a work population of size class 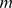 is plotted as a density plot. The data were obtained from the Urban Transportation Census (UTC) commute data (Ministry of Land, Infrastructure, Transport and Tourism, The 10th Urban Transportation Census Report, 2007; in Japanese).

Using these data, the daily commute movement of each individual in our individual-based model is implemented in the following way. Individuals are assumed to travel by commuter train from their residence (home population) to their workplace/school (work population), stay at their workplace/school during the day, and return from their workplace/school to their residence by the commuter train again and remain at their residence during the night (Figures 2A–B). Since the commuting data are acquired from commuter pass, this recurrent pattern only represents the commuting data of workdays. Weekend travels are generally irregular without specific patterns and we do not have the relavent data. However, since we have 2 holidays out of 7 days in a week and much less traffic flows compare to workdays, we have neglected this effect of weekends for simplicity.

Let 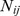 be the number of individuals commuting from station 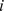 of their residence to the station 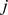 of their workplace/school. In other words, 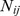 is the number of commuters between the home population 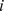 and work population 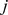 and is calculated from the UTC commute data. From 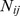, we define 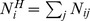 and 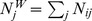, which respectively gives the number of commuters (traffic volume) that use station 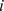 as the home population, and the number of commuters that use station 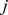 as the work population, respectively. It should be noted that the same station could be the home population for one commuter and the work population for another. Therefore, station 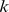 is characterized by 2 sizes of commuter groups, which are the size 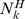 of the home population that represents the residential population, and the size 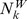 of the work population that represents the working/studying population. Here, we defined the commuter sizes in terms of those in the UTC sample (of the total of 139,841) i.e., the sample constituted about 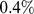 of the residents of the Tokyo metropolitan area.

### Individual-based model: Epidemic dynamics

Infection of a disease is assumed to occur as follows: a susceptible individual making contact with an infectious individual in the residence area and/or the workplace/school area is infected according to the infection rate of the disease. Once infection has occurred, the newly infected individual continues commuting as an infectious host in an asymptomatic state. As the disease became symptomatic, the infected individual will stop commuting and rests within his or her household until recovery. After the recovery, the recovered individual will acquire immunity against the disease and resume commuting again. Therefore, a commuter's epidemiological status could take 1 of 3 states: susceptible (S), infected and infectious (I), or removed from the commute network for recuperation or recovered from the infection with immunity (R). Although the recovered individual resumes commuting, he or she plays exactly the same role in the epidemic dynamics as those who are still resting and not commuting. Each individual is assigned to 1 of the 3 states at each moment. Accordingly, the number of commuters between home population 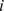 and work population 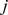 at time 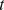 is decomposed to 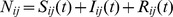, where 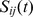, 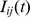, and 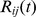 denote the number of individuals in state S, I, and R, respectively. As we are interested in the spread of infectious disease within a single season, the birth and death of individuals are neglected. Therefore, the total number of commuters 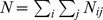 remained constant throughout the calculation. Furthermore, assumptions were made, as follows: (i) each individuals will not change their daily commute path within a season, i.e., each 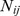 is also kept constant; (ii) infection only occurs within each subpopulation (home populations and work populations), i.e., infection occurring on the commuter train is neglected; (iii) as the day and night populations of the same location are completely separate in our model, there is no contact between an individual who is part of the home population as a resident at night and another who is part of the work population of the same area during the day; and (iv) During the recuperation period at their households, the infection within their household does not occur.

The simulation was individual-based, in which commuter movements and infection processes of each of the 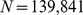 individuals are kept tracked. The actual population in this area is approximately 100 times larger than this; however, we avoided extrapolating to the actual population numbers owing to the high computational cost it would have entailed. Every day, each individual commute from his or her home population to his or her work population, work/study during the daytime and return to their residence area at nighttime. We denote the number of infectious individuals within home population 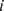 and work population 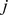 on day 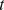 (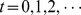) by 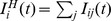 and 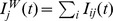, respectively. For a susceptible individual commuting from home population 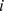 to work population 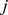, the mean number of contacts with an infectious individual on each day is considered to be 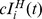 for the home population and 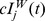 for the work population, where 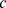 is the constant contact rate. Then assuming that the infection will occur according to the Poisson process with the expected number of newly infections per day being 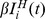 and 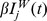 for home and work population, respectively. Here, 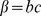 is the rate of disease transmission or the infection rate from a single infectious host per day (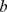: rate of disease transmission per single contact with an infectious individual). Given this the probability that an individual gets infected during day 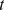 becomes 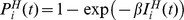 for home population 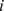 and 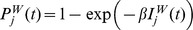 for work population 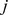. The probability that the infectious individual to be removed from the commute network on each day is 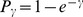, where 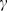 is the rate of transition from the asymptomatic to the symptomatic state. Accordingly, 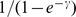 is the average duration of the asymptomatic state. The following transition diagram schematically represents the overall infection process for each individual living in the 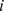-th population and commuting to the 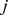-th population (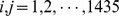):
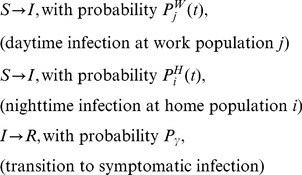
(1)


In the simulation, each individual's epidemiological state changes stochastically according to the transition diagram (1). This process is repeated until the total number of infectious individuals becomes zero.

The epidemic process is initiated by introducing a single infected and infectious host into the commute network. The sizes of the home and work populations of the initially infected host turn out to play a critical role. To examine this dependence on the population size of the initial infected site, we first group all home and work populations according to their logarithmic size. After choosing the size classes of the home and work populations of the initially infected host, we chose the initial infected host's home and work populations randomly from each size class. Hereafter, the home and work populations of this initial infected host are called the initial home population and the initial work population, respectively. The size of the largest work population exceeded 5,000 (e.g., Shinjuku station with a working/studying population of 5,412), but that of the largest home population was below 1,000 (e.g., Oizumi-gakuen station with a residential population of 853). For each epidemic parameter, 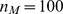 Monte Carlo simulations are performed. Throughout the paper, the average duration of the asymptomatic state is fixed to be around two 2 days [21], so a transition rate of 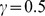 was used in all calculations.

The spread of infectious disease over the commute network is evaluated by the number of infected local populations, that is, the number of local populations in which at least 1 individual became infected during the infection process. Among 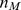 independent replicates of a Monte Carlo simulation with fixed epidemic parameters and initial conditions but different random number seeds, the number of infected local populations shows a bimodal distribution in both the home and work populations. For each home or work population, the distribution of the number of infected local populations has a peak at 1 (i.e., the population with the initially infected individual) and another peak at more than several hundred of the total of 1,435 local populations. As the widths of both peaks had relatively narrow widths (the order of several tens), we can unambiguously classify each run of the Monte Carlo simulation as an initial extinction or global epidemic.

### Population size class model

In order to analyze the simulated results from the individual-based model, we further construct a simplified epidemic model in which the connectivity between the different population size classes of home and work populations is set to be consistent with the UTC data (see below). Here, all characteristics of each local population (e.g., geographic position and position within the commute network) and the details of the commuter flow between the local populations are ignored. Accordingly, in this model, local populations with different locations but within the same population size class are treated equally (Figures 2 B–C). We henceforth call this model the population size class model (PSCM), and a detailed description is given in Supporting Information S1.

We use the previously defined size class distributions for the home and work populations for the PSCM (see section Individual-based model: Epidemic dynamics). Let 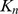 be the representative population size of the 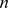-th population size class. The group of commuters traveling between the 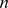-th home population size class and 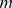-th work population size class is referred to as the commuter population of the 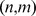-th size class, and the number of such commuters is denoted by 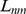. The actual values of 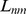 utilized in the PSCM are calculated from the UTC sample data and given in Figure 2D. For the given data for 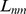, the probability of a global epidemic is calculated from the stochastic PSCM and the epidemic dynamics is calculated from the deterministic PSCM. The usage of UTC sample data in PSCM will give the same results as the calculation based on actual population for a suitable choice of rate of disease transmission value giving the same basic reproduction ratio (See supporting information for details).

In the stochastic PSCM, a global epidemic is defined as the case in which the infection never dies out during the branching process. We then calculate the probability that an infection starting from an 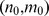-th commuter population size class will eventually be extinct (i.e., the extinction probability of the infection). The details of the mathematical formulation of the stochastic PSCM are given in Section B of Supporting Information S1.

The final size of the global epidemic (the fraction of individuals among the total population who eventually became infected) and the time of the epidemic peak (the time at which the total number of infectious individuals attains its peak) are obtained using the deterministic PSCM, which is formulated as a system of difference equations (Equation (7), (8) and (9) in Section C of Supporting Information S1). Furthermore, the epidemic dynamics within each local population, such as the final size of the epidemic and time until the epidemic reaches each local population, are also investigated using this model. Especially, the explicit form of the arrival time of the epidemic is given. The details of the mathematical formulation of the deterministic PSCM are given in Section C of Supporting Information S1.

### Random reconnection model

In addition to the PSCM, an individual-based model simulation using the commute network data, which is constructed based on the random reconnection model (RRM) is also studied to investigate the roles of the geographical and topological positions of station nodes within the network. The RRM is constructed from the original commute data for the Tokyo metropolitan area by randomly reconnecting the commuter flows while retaining the connectivity between the population size classes. Therefore, the number of commuters 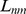 within the commuter population 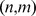-th size class in the RRM is identical to that from the original UTC data (Figure 2D). The commute data for the RRM is constructed as follows: (i) a pair of individuals is randomly chosen from the 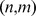-th size class of commuter populations (i.e., from the set of individuals whose home and work populations belongs respectively, to the 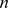-th and the 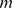-th size class), and their work populations are changed; and (ii) this process is repeated until all (or all but one, if the total number of individuals in the size class is odd) of the individuals are chosen. This process randomly reconnects the original commuter flows without changing the connectivity between the population size classes. The original geographical structure of the commute network is completely lost in the RRM, as in the PSCM. The probability of a global epidemic, final size of the global epidemic, and time of the epidemic peak are obtained from the IBM simulation using these data and compared with those from the simulation on the basis of the original commute data.

## Results

### Probability of a global epidemic

**Figure 3:**
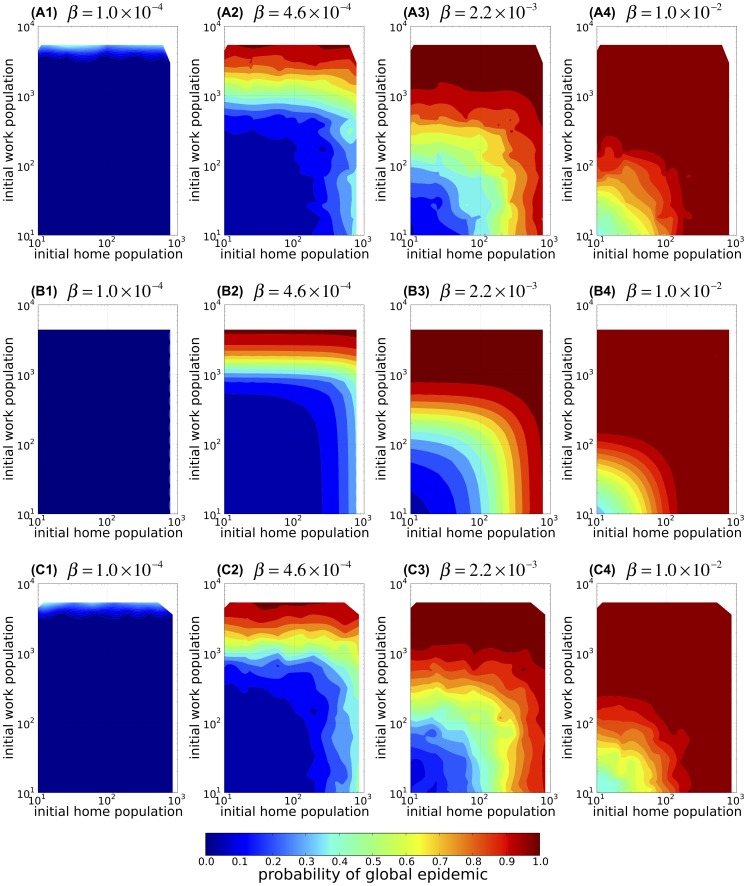
The probability that a single infected host causes global epidemic. The probability of a global epidemic, 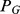, as a function of the home population size (horizontal axis; initial home population) and work population size (vertical axis; initial work population) of the initially infected host for various infection rate 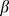. (A1–4) The results of the individual-based model (IBM) simulations of the spread of infectious disease over the commute network of the Tokyo metropolitan area which starts with a single infectious individual commuting from a randomly chosen home population to a randomly chosen work population. Each panel corresponds to a different infection rate, and the population sizes are plotted on logarithmic scales. (B1–4) The corresponding results obtained using a branching process formula in the population size class model (PSCM). (C1–4) The corresponding results of the IBM simulations using the random reconnection model (RRM). In each panel, the contour plot represents interpolation of the results calculated using the data of 184 combinations of initial home and work population sizes.

The probability of a global epidemic 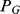 is defined as the fraction of the independent runs of the Monte Carlo simulation in which a global epidemic occurred (i.e., 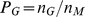, where 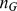 denotes the number of global epidemics observed within 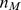 runs of the Monte Carlo simulation; refer to Section Individual-based model: Epidemic dynamics for a more detailed definition of a global epidemic). The probability of a global epidemic 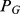 observed in the IBM simulation for a given infection rate and sizes of the home and work populations of the initial infected host is plotted in Figure 3A. When the infection rate is low, initial extinction of the disease prevails over the global epidemic in an initially infected population of any size (Figure 3A1). However, when the infection rate is sufficiently high (Figures 3A2–4), 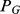 increases with the sizes of either initial home or work population.

The probability of a global epidemic calculated by the stochastic PSCM is plotted in Figure 3B. A comparison of Figure 3B with Figure 3A highlights the close resemblance between the results of the PSCM and those of the IBM. Therefore, despite the greatly simplified assumptions of the stochastic PSCM, which ignore the details of the connections between any specific pair of populations, the resulting model excellently explains how the probability of a global epidemic calculated from the branching process recurrence formula (Equation (4) in Section B of Supporting Information S1) depends on the infection rate and the sizes of the initial home and work populations. Therefore, unsurprisingly, the results of the randomly reconnected IBM simulation (RRM) that uses the actual connectivity between size classes from the original commute network data are similar to those of the original IBM simulations (compare Figure 3A with Figure 3C).

**Figure 4:**
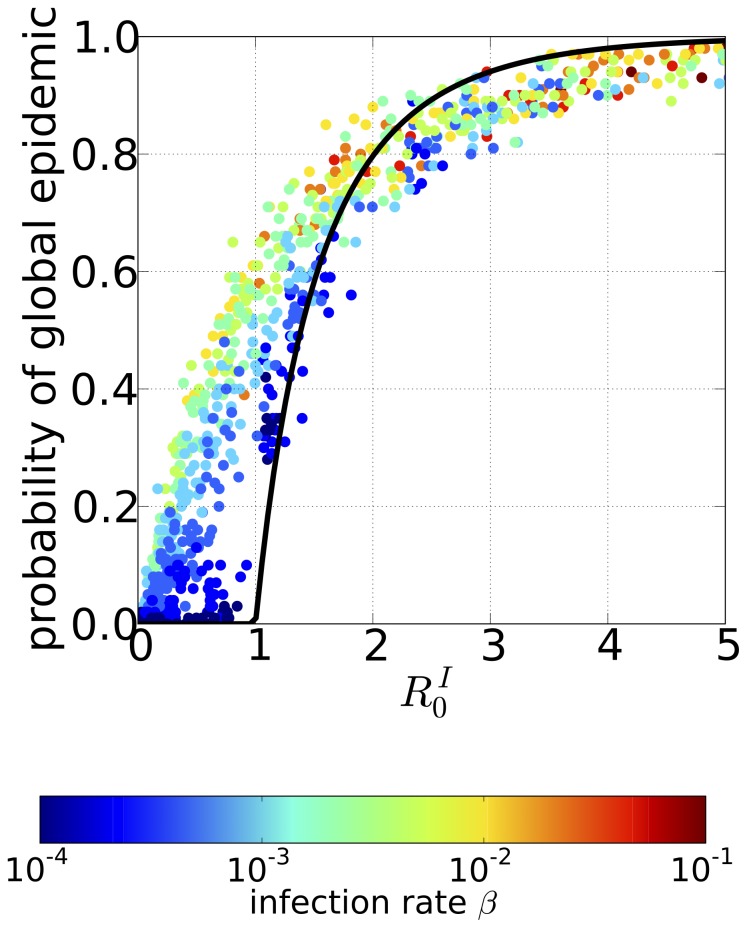
The probability of global epidemic as a function of the basic reproductive ratios in the initially infected home and work populations. The probability of a global epidemic observed in the individual-based model (IBM) simulations based on the commute network data for the Tokyo metropolitan area plotted as a function of 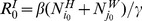, where the independent variable is the sum of the single population basic reproduction ratios of the initial home and work populations (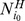 and 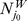: home and work population sizes of the initially infected individual, respectively). Each point corresponds to a different set of epidemic parameters, and the color represents the infection rate 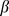. Black line, the probability of a global epidemic in the single population model with population size 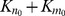, i.e., that from 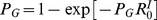 (main text for details).

The invasion condition can further be simplified by a careful inspection of the branching process recurrence formula of the stochastic PSCM. We find that the probability 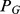 of a global epidemic defined in the branching process for the initial spread of the infection can be approximated as (Equation 6 in Section B of Supporting Information S1):
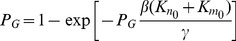
(2)which depends only on the sizes 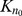 and 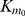 of the home and work populations, respectively, to which the initially infected host resides (the black line in Figure 4). As the basic reproduction ratio of a single homogeneous population with the population size 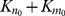 is given by
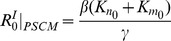
(3)the probability of a global epidemic 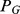 in equation (2) coincides with the probability that the infection will occur in a single isolated population of size 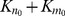 [18], [22]. Figure 4 shows the probability of a global epidemic 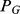 observed in the IBM simulations (dots) for various infection rates 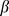 (different colors) and home and work population sizes of the initially infectious host 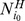 and 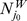. The probability of a global epidemic 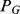 for different combinations of 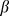, 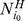, and 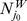 can be plotted against 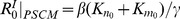, resulting in a single but indistinct band.

In summary, the following conclusions can be drawn for the probability of a global epidemic: (i) the probability of a global epidemic is mainly determined by the connectivity between population size groups; information on the connections between a specific pair of local populations is dispensable for the prediction of whether or not the disease will spread globally or not; and (ii) the sizes of the home and work populations of the initially infected individual contributes greatly to the probability of a global epidemic. This is because the invasion of the entire commute network relies mainly on whether the disease can invade the initial home and work populations.

### Extent and speed of disease spread

The overall damage of a global epidemic in the Tokyo metropolitan area is measured by the final size of the global epidemic, which is defined as the fraction of infected individuals within the total population. To investigate how quickly does the disease will spread out, we also examined the time of the epidemic peak, which is defined as the time until the total number of infectious individuals attains its maximal value. We used these parameters to evaluate the extent and speed of the spread of the disease.

**Figure 5:**
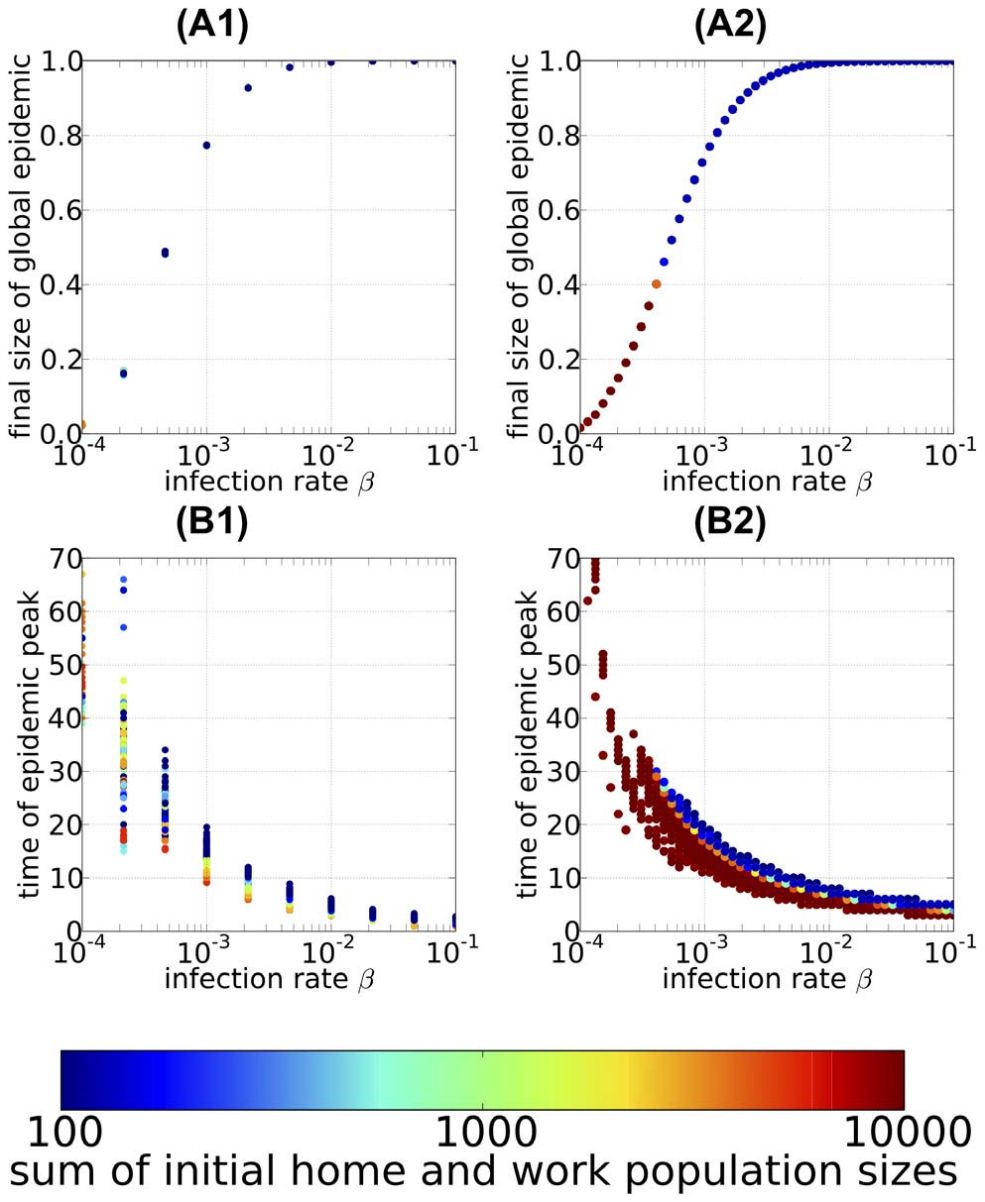
The final size and the peak time of global epidemic. The final size of the global epidemic (A) and the time until an epidemic initiated by a single host reaches its peak (B) plotted against the infection rate 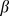. (A1) and (B1): results observed in the individual-based model (IBM) simulations; each point gives the Monte Carlo ensemble average value corresponding to different epidemic parameters, and the color indicates the sum of the sizes of the initially infected home and work populations. Here, the cases for initial extinction of disease are excluded from the ensemble. (A2) and (B2): corresponding results from the population size class model (PSCM) calculations.

The final size of the global epidemic as a function of the infection rate is shown in Figure 5A1; each point corresponds to a different set of initial home and work populations and represents the mean value across the ensemble of Monte Carlo simulations in which a global epidemic occurred. In Figure 5A1, the final sizes of the global epidemics for a given infection rate are concentrated to a single point, which implies that once a global epidemic is underway, its final size is almost independent of the initial home and work population sizes. This contrasts sharply with our result for the probability of a global epidemic, which is quite sensitive to the sizes of the initial home and work populations. For 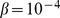, although a global epidemic did occur, its final size remained very low and only a small percentage of the population is infected. As the infection rate increases, the final size of the global epidemic increases until it approaches 1, meaning that almost all individuals in the population are infected, at around 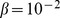. As the figure shows, the infection rate had a threshold value 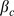, approximately at 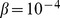, below which global invasion of the epidemic could not occur. At this critical value of the infection rate, the local basic reproductive ratio (i.e., the single population basic reproduction ratio 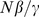) in the Shinjuku station area (the largest work station) is 1.083, which is only slightly greater than 1. In contrast, the local basic reproductive ratio for the critical infection rate in the Oizumu-gakuen station area, the largest home population, is much lower than 1 (0.17). When we considered the mean rather than the largest population sizes, the local basic reproductive ratio for the critical infection rate was just 0.217 in the mean work population and was even smaller, 0.0546, in the mean home population. This suggests that a few very large work populations can play a pivotal role in an epidemic outbreak in the Tokyo metropolitan area.

The time to the epidemic peak as a function of the infection rate is shown in Figure 5B1; each point corresponds to a different set of initial home and work populations and represents the mean value across the ensemble of the Monte Carlo simulations in which a global epidemic occurred. The time to the epidemic peak is longest when the infection rate is close to the threshold value 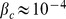 at which the final size of the global epidemic began to increase from 0; the time to peak then decreased monotonically as the infection rate increases. In contrast to the final size of global epidemic, the time to the epidemic peak retains a significant dependence on the initial home and work population sizes at small infection rates, reflected in the plot as the wide distribution of the time to the epidemic peak.

The final size of the global epidemic and the time to the epidemic peak obtained from the deterministic PSCM are shown in Figures 5A2 and 5B2, respectively. Here, each point corresponds to a different set of initial home and work populations, and is plotted against the infection rate. Both values exhibit characteristics similar to the results of the IBM simulation, and comparison between the models show that the PSCM could approximate both the final size of the global epidemic and the time to the epidemic peak. Moreover, the final size of the global epidemic shows little dependence on the sizes of the initial home and work populations (shown analytically by Equation (14) in Section C of Supporting Information S1), whereas the time to the epidemic peak shows substantial dependence on the sizes of the initial home and work populations. Another IBM simulation with random reconnection of the local populations using the RRM is performed and yield a final size of the global epidemic and time to the epidemic peak almost identical to those obtained from the IBM using the original commute network data (data not shown owing to the perfect overlap with the original data).

In conclusion, we find that: (i) the final size of the epidemic and time to the epidemic peak are mainly determined by the connectivity between the population size classes, as both the PSCM and the RRM, which completely ignore the population-to-population connectivity, can reproduce the results of the original IBM simulations; (ii) the final size of the global epidemic is determined almost solely by the infection rate. Under the threshold infection rate for disease invasion, only a few largest work population areas have local basic reproductive ratio greater than 1; (iii) the time to the epidemic peak is quite sensitive to the sizes of the initial home and work populations of the initially infected individual.

### Epidemic dynamics within each local population

The epidemic dynamics within each local population is evaluated by the final size of the local epidemic and by the time until the epidemic reached the local population (the arrival time). The final size of the local epidemic within each local population is defined as the fraction of commuters out of the local population who eventually became infected during the infection period (i.e., 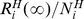 and 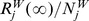 for the 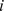-th home population and 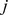-th work population, respectively). The arrival time of the epidemic is defined as the timing that the infected commuters first appear in each local population after the initial infection started (i.e., the arrival times 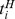 and 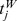 of the epidemic in the 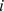-th home population and 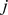-th work population, respectively, are given by 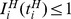 and 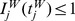, i.e., where the time until the number of infected individuals reaches a predetermined small number, which is 1 in our IBM simulation and about 100 in the actual population). For each home or work population, the final size and the arrival time of the local epidemic are calculated as the mean across the ensemble of Monte Carlo simulation runs in which a global epidemic occurred and at least 1 infected individual appeared in the local population.

**Figure 6:**
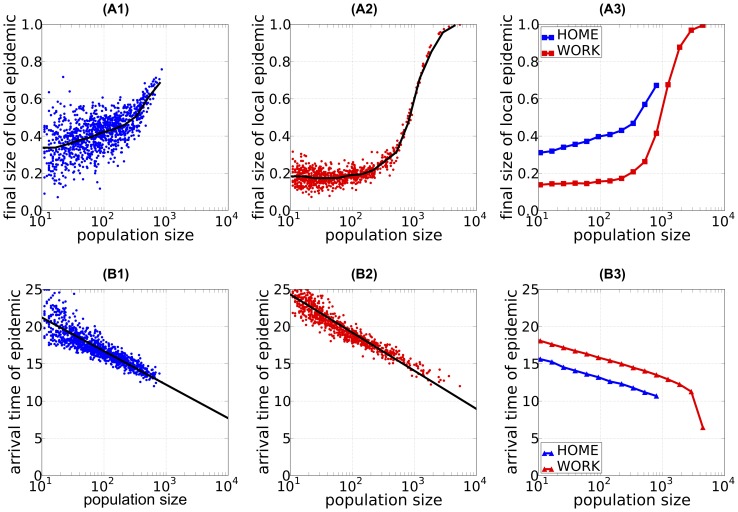
The final size of epidemic and the arrival time of epidemic at local populations. The final size of the local epidemic (A) and the time until the infected individuals first appear in the local population (B) (i.e., the arrival time of epidemic) plotted against the local population size. The population size is on a logarithmic scale. (A1–2) and (B1–2): results of the individual-based model (IBM) simulations; each point (dots) gives the mean value of the Monte Carlo ensemble averaged over 100 Monte Carlo runs for each local population, and the blue and red dots correspond to the results for the home and work populations, respectively. The black lines in (A1–2) give the mean value of the final size of the local epidemic for each population size class. The black lines in (B1–2) represent the regression line of the arrival time of the epidemic in the local population versus the logarithm of the population size. The regression line for the arrival time 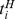 in the 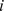-th home population with population size 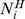, 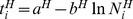, was highly significant, with a P-value of 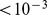 in the 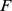 test (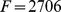 with the degrees of freedom (1, 1084)), 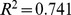. The estimated intercept 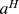 and slope 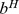 and their 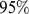 confidence intervals (CIs) are 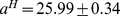 (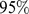 CI) and 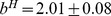 (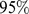 CI). The same was true for the arrival times in the work population; the regression 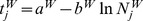 was highly significant (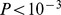, 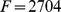 with 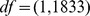), with estimated intercept and slope 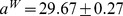 (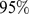 CI) and 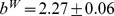 (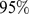 CI), respectively. (A3) and (B3): corresponding results obtained from the population size class model (PSCM); the blue line shows the result for the home population and the red line the result for the work population (refer main text for details). The infection rate was 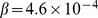. A person commuting from “Gyotoku” station to “Aoyama-itchome” station was designated the initially infectious individual.

The final size of the local epidemic within each population is shown in Figures 6A1–2; each dot represents the mean value across the ensemble of the Monte Carlo simulations in which a global epidemic occurred. In both home and work populations, larger populations have larger final local epidemic sizes. In sufficiently large work populations (exceeding 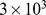 commuters), almost all of the commuters within the local home population are infected, whereas in sufficiently small work populations (less than 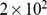 commuters), only approximately 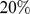 of the commuters within the local work population are infected. The final size of the local epidemic is larger in home populations than in work populations of the same size. Moreover, we found that the final size of the local epidemic in both home and work populations depends strongly on the infection rate and the size of the local population but negligibly on the sizes of the initial home and work populations. The deterministic PSCM exhibited qualitatively similar dependencies, although the final sizes of the local epidemics are slightly smaller (Figure 6A3). Therefore, the local population size alone is sufficient to predict the total damage caused by an epidemic in a local population.

**Figure 7:**
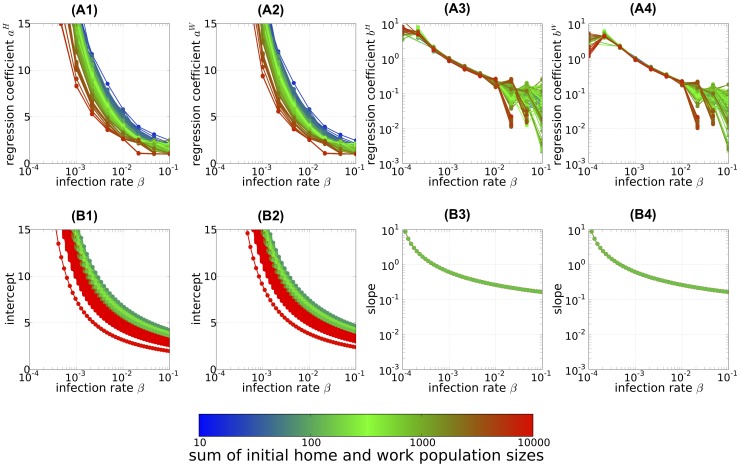
The intercept and slope of the linear relationship between the arriving time of epidemic and logarithmic local population size. The intercept (A1–2) and slope (A3–4) of linear regression line for the arrival time of epidemic, and the corresponding results calculated from the exponential growth approximation of the linearized population size class model (B1–2) and (B3–4), respectively (see main text for details of approximation) are shown. The results are plotted as the functions of infection rate and the color of each line indicates the sum of the population sizes of the initially infected home and work populations. (A1–4): regression coefficient statistically estimated from the results of the individual-based model (IBM) simulations. (A1) and (A2): estimated intercepts 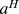 and 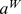, respectively. (A3) and (A4): estimated (sign reversed) slopes 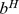 and 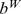, respectively. All regressions were statistically significant according to the P-value of the regression coefficient (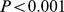).

The arrival time of the epidemic in each local population is shown in Figure 6B1–2; each point represents the mean value across the ensemble of Monte Carlo simulations in which a global epidemic occurred. Similar results obtained by deterministic PSCM are given in Figure 6B3. For both home and work populations, a larger population size is associated with faster arrival time of the epidemic. Moreover, the arrival time of the epidemic clearly depends on the local population size i.e., it decreases in a linear manner on a semi-logarithmic plot: 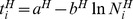 and 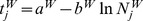 for the 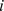-th home population and 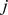-th work population, respectively. The regression coefficients are: for home populations, intercept 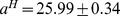 (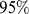 confidence interval, CI) and slope 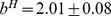 (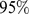 CI) (coefficient of determination: 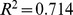); and for work populations, intercept 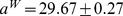 (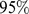 CI) and slope 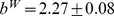 (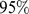 CI) (coefficient of determination: 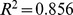). Roughly speaking, increasing the local population size 3-fold causes the epidemic to arrive 2 days earlier, and infection of a work population lags that of a similar-sized home population by approximately 4 days. These results show a strong statistical relation between the arrival time of an epidemic in a local population and the size of that population. Figures 7A1–2 and 7A3–4 show how the intercepts (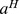 and 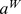) and the slopes (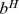 and 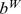) of the regression depend on the infection rate and the size of the initially infected population. In both home and work populations, the intercept decreases as the infection rate increases (Figure 7A1–2). The slope also decreases with the infection rate (Figure 7A3–4), indicating that the dependence on the local population size is weaker when the infection rate is sufficiently large. The intercepts (Figure 7A1–2) but not the slopes (Figure 7A3–4) depend on the sizes of the initial home and work populations. The intercept depends especially on the sum of the population sizes of initial home and work populations; as this sum increases, the intercept decreases, signifying an earlier overall arrival time of the epidemic (Figure 7A1–2).

This logarithmic dependence of the arrival time of the epidemic on the local population size can be explained by the deterministic PSCM (Figure 6B3). When we linearize the system of difference equations of the deterministic PSCM (Equation 7, 8, and 9 in Section C of Supporting Information S1) around a disease-free state and assume that the largest eigenvalue component dominates in the initial exponential phase of the system, we see that the arrival time of the epidemic in the 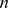-th home (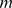-th work) population size class 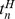 (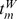) is given approximately by
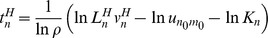





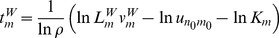
(4)


(Section C of Supporting Information S1 for the derivation). We define the arrival time of the epidemic in the deterministic PSCM as the time at which the number of infected hosts reaches a predetermined small number (Equation 16 in Section C of Supporting Information S1). Here, the largest real eigenvalue of the system of linearized difference equations is denoted as 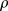 and the 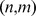-th elements of the corresponding right and left eigenvectors as 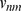 and 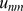, respectively. 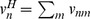 (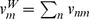) gives the relative fraction of the 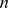-th home (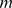-th work) population size class in an exponentially growing infected population, and 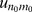 gives the “reproductive value” [23] of the initially infected commute population size class. We compared the results of the numerical calculation of the system of difference equations of the deterministic PSCM (Equations 7, 8, and 9 in Section C of Supporting Information S1) with formula 4 and found that they largely agree. The resulting arrival time of the epidemic is shown in Figure 6B3. This model also shows a logarithmic dependence, although the arrival time of the epidemic is systematically earlier compare to that of the stochastic IBM simulation. The discrepancy may be ascribed to failure of stochastic colonization of local populations in the early stage of the epidemic. In spite of this discrepancy, the deterministic PSCM explains the logarithmic dependence on the local population size and its epidemic parameter dependence quite well, as shown below.

In addition to the logarithmic population size dependence discussed above, we extracted another dependency from the explicit formula Equation 4 for the arrival time of the epidemic. The arrival time of the epidemic becomes earlier as the logarithmic odds ratios 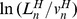 and 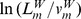, the actual number of commuters in each population size class to the number expected in an exponentially growing infected population, increases. The reproductive value of the initially infected population size class also helps to shorten the arrival time. We were able to obtain these values easily from the population size class model of the commuting population of the metropolitan area and the epidemic parameters (i.e., the infection and removal rates). Figures 7B1-2 and 7B3–4 summarize how the intercept and the slope of the arrival time of epidemic depend on the population size; the results can be compared with Figures 7A1–2 and 7A3–4 for the IBM calculation. The analytical predictions plotted in Figures 7B1–2 and 7B3–4 were given by 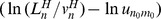, 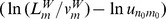 (intercept) and 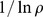
(slope) for the home and work populations, respectively. Here, we approximated by attributing the population size class dependence only to the third factor in Equation 4, the logarithmic population size factor (i.e., 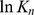 and 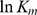; Section C of Supporting Information S1 for the justification of this approximation). The parameter dependence of the intercepts and slopes was quite similar to that observed in the IBM simulations (Figure 7A1–4) and Equation 4 (Figure 7B1–4). The effect of the initial population size on the intercepts is apparent in their dependence on the sum of the initial population sizes (different-colored lines in Figures 7A1–2 and 7B1–2). The slope, in contrast, exhibited no initial population dependence; the reason for this absence is obvious from the explicit form of Equation 4, as the eigenvalue and eigenvectors derived from the population size class model and do not depend on the initial condition. In summary, the overall results of the deterministic PSCM are consistent with the results of the IBM calculation except that the former yielded a consistently earlier arrival time of the epidemic.

## Discussion

In this study, we analyzed the epidemic process over the commute network in the Tokyo metropolitan area. We first used actual commute data to simulate the spread of infectious disease in an individual-based model and then constructed a simple mathematical model (the stochastic and deterministic PSCM) to gain further understanding of the epidemic dynamics. In particular, we examined which aspects of the commute network structure affect the global epidemic dynamics by calculating the probability of a global epidemic, the final size of the global epidemic, and the time to the epidemic peak. We also investigated the properties of the epidemic within each local population by examining the final size and arrival time of the epidemic. Finally, we compared the results of the IBM with those of the PSCM, which reveal that both the stochastic and deterministic PSCM capture the essence of the epidemic process except that the epidemic reached each local population earlier in the PSCM than in the IBM.

Inspection of the results obtained using the IBM along with the PSCM-based analysis led us to the following 3 major conclusions: (i) when an initially infectious individual appears in the area, the probability that a global epidemic will occur is determined mainly by the infection rate and the population sizes of the individual's home and work populations, indicating that the geographical location within the commute network have only a minor effect; (ii) the final size of the global epidemic and time to the epidemic peak depend greatly on the connectivity between the population size classes but, again, are insensitive to the connection geometry of the individual populations; and (iii) the arrival time of the epidemic in a local population shows a simple dependency, decreasing linearly with the logarithmic size of the population.

The first and second conclusions, concerning the insensitivity of the epidemiological process to the specific geometry (detailed information on the number of individuals commuting between a specific pair of local populations), can be ascribed to the existence of very large work populations (Figure 1). Although the commuting population in the Tokyo metropolitan area forms a complex network structure, the presence of these large hubs enables most of the individuals to contact each other either directly or indirectly (i.e., through another individual contacting both of them) within their shared large work populations. This characteristic makes the epidemiological process along the commuter network almost independent of the network's spatial configuration. In other words, any spatial pattern of the epidemiological process is easily lost once the disease reaches one of the hub populations.

To the best of our knowledge, the third conclusion, that there is a very clear logarithmic relation between the local arrival time of the epidemic and the local population size, has not previously been reported. We should however note that Gautreau et al.[24]'s theoretically analyses on the arrival time of epidemic to each sub-population might be related to our finding, though both the derived formula and the ways they are derived are quite different between ours and theirs. They derived an approximate relationship between specific network characteristics to the arrival time of epidemic, where the average arrival time is given as the minimum, along all possible paths connecting the primarily infected sub-population and the focal sub-population, of weighted sum of logarithmic sub-population sizes along the path. In our formula only the sub-population size at the end-point of the paths influences the arrival time, and this can well explain the logarithmic population size dependence of arrival time in our Monte Carlo simulation. It is left for a future study to examine whether or not this insensitive dependence of intermediate sub-populations along paths on the arrive time is specific to our case of Tokyo metropolitan commute network, where we have very large ‘hub’ work populations along the JR Yamanote line.

This logarithmic population size dependence of the arrival time of epidemic, suggests that when monitoring a metropolitan area for disease invasion, it would be most effective to focus on large-sized local populations in which the disease is likely to arrive earlier than in smaller populations. We can then predict the time at which the disease will appear in each local population after its initial detection in a large population by determining the ratio of the sizes of the initially infected and naive local populations. The explicit form of the arrival time of the epidemic in Equation 4 warrants further discussion of the arrival time of the epidemic. The reason why we observed the logarithmic local population size dependence in the arrival time of epidemic can be ascribed to our definition of the arrival time of the epidemic as the time until the number of infected hosts reaches a predetermined value (Equation 16 in Section C of Supporting Information S1). However, if we defined the arrival time of the epidemic as the time until a certain fraction of the local population becomes infected, the dependence on the logarithm of the population size would disappear. Furthermore, if the transmission dynamics obey the density-dependent transmission, as adopted in this paper and in many models of contagious diseases except for vector-borne diseases, which are usually modeled as frequency-dependent transmission)[25], the time at which the number of infected cases reaches a preset value determines the later pace of the epidemic dynamics in the population. If this holds true in practical applications, we would expect the arrival time in a local population to depend on the logarithm of the population size.

Our results show that once a global epidemic does occur, its final size becomes independent of the location at which the infection initiated. However, because the probability of a global epidemic increases with the home and work population sizes of the initially infected hosts, prophylactic vaccination would be most effective if applied to individuals who live in large home populations and commute to large work populations. The clear relation between the arrival time of the epidemic and the local population size suggests an effective strategy for local intervention. If early cases of infection were detected in large local populations, our formula would enable us to predict the expected arrival times of the epidemic in smaller local populations. If the infection rate is low but sufficient to sustain a global epidemic, a population approximately one-third the size should experience disease arrival approximately 2 days later, on average (Figure 6B). This knowledge would thus enable rapid intervention against the disease invasion prior to its arrival. However, the time available for such preparations decreases as both the infection rate and the population size of the local home and work populations of the initially infected individual increases.

Several factors are not included in our models. One of these is the spread of infection within the commuter trains during the commute. The importance of transmission within commuting subway has been addressed by Cooley et al.[26]. However, as we are interested in how the network structure of the commuting population would affect the epidemic dynamics, the inclusion of intra-train infection would have drastically increased the number of combination of patterns of contact between the commuters and making the theoretical analysis intractable. However, as the commuter trains in the Tokyo metropolitan area are overcrowded during peak commuting hours, consideration of this effect are a necessary challenge for future work. Another factor that we do not include is the effect of the non-commuting portion of the home population; this limitation arises from the fact that the UTC was intended as a transportation survey and did not include information about the non-commuting population. Although this omission might quantitatively affect the results of our simulations, we believe that our model partially accounts for this effect by including the night spread of infection within the home populations.

## Supporting Information

Supporting Information S1 **Description of population size class model (PSCM).** Formulation of PSCM from the commute network data of Tokyo metropolitan area is given in Section A. The stochastic version of PSCM to analyze the probability of a global epidemic is given in Section B. The deterministic version of PSCM to analyze the final size of the global epidemic, the time until the global epidemic attains its peak, the final size of the local epidemic, and the arrival time of the epidemic in each local population is given in Section C.
(PDF)
