# Correction: Epidemic process over the commute network in a metropolitan area

**DOI:** 10.1371/journal.pone.0132238

**Published:** 2015-07-08

**Authors:** Kenta Yashima, Akira Sasaki

In the Results section, there is an error in Eq 4. The quantity in parentheses is incorrect. Please view the complete, correct Eq 4 here:

$$t_{n}^{H}=\frac{1}{\ln\;\rho }\;(\ln\frac{L_{n}^{H}}{v_{n}^{H}}-\ln\;u_{n_{0}m_{0}}-K_{n})$$

$$t_{m}^{W}=\frac{1}{\ln\;\rho }\;(\ln\frac{L_{m}^{W}}{v_{m}^{W}}-\ln\;u_{n_{0}m_{0}}-K_{m})$$
