# Supplementary material for: Epidemic Process over the Commute Network in a Metropolitan Area
Source: PLoS One. 2014 Jun 6;9(6):e98518. doi: 10.1371/journal.pone.0098518 (PMC4048205; doi:10.1371/journal.pone.0098518)
Supplement: Supporting Information S1 — Description of population size class model (PSCM). Formulation of PSCM from the commute network data of Tokyo metropolitan area is given in Section A. The stochastic version of PSCM to analyze the probability of a global epidemic is given in Section B. The deterministic version of PSCM to analyze the final size of the global epidemic, the time until the global epidemic attains its peak, the final size of the local epidemic, and the arrival time of the epidemic in each local population is given in Section C. (PDF) [file pone.0098518.s001.pdf]

Supporting Information S1

A population size class model

The population size class model (PSCM) is formulated in order to analyze the results of the simulations of the epidemiological dynamics of the commute network in the Tokyo metropolitan area using our individual-based model (IBM). This section describes how we defined the PSCM (Section A); used the stochastic version of the PSCM to analyze the probability of a global epidemic (Section B); and used the deterministic version of the PSCM to analyze the final size of the global epidemic, the time until the global epidemic attains its peak, the final size of the local epidemic, and the arrival time of the epidemic in each local population (all Section C). At the final part, we particularly focus on the clear relationship between the arrival time of the epidemic at a local population and its logarithmic population size dependence that are observed in the IBM simulations.

This model is based on the commute network data for the Tokyo metropolitan area but incorporates only the connectivity between the different population size classes of the home and work populations. This was accomplished by classifying all home and work populations into population size classes. We use L_{nm} to represent the number of commuters between the n-th home population size class with representative population size K_n and the m-th work population size class with representative population size K_m (Figure 2D). Given this, the number of individuals in the n-th home (m-th work) population size class is given by $L_n^H \equiv \sum_m L_{nm}$ ($L_m^W \equiv \sum_n L_{nm}$). The values of L_{nm} are obtained from the sample data of UTC [1] just like the IBM simulations. Hereafter, for simplicity, we refer to the group of commuters traveling between the n-th home population size class and the m-th work population size class as the commuter population of the (n, m)-th size class. We then define the epidemiological dynamics within these groups of commuters (PSCM). For a given set of L_{nm}, the probability of a global epidemic is calculated using a branching process [2] (Section B), and the other epidemiological properties for the deterministic PSCM are derived (Section C). Only the connectivity via commuting flows between the above-defined population size classes, out of all of the characteristics of the commute network, is used in the PSCM. Therefore, we ignore all of the other details, such as the geographical locations of the local populations and the complicated connectivity properties that could not be summarized by the commuter flows between the size classes. Nonetheless, as stated in the main manuscript, such a simple model could explain a great deal of the results obtained from the individual-based model.

Numerical burden to calculate the PSCM is relatively small compare to IBM simulation, thence it seems reasonable to use the actual population data of Tokyo metropolitan area, then to use the sample data of UTC. However, when each population size is scaled-up to the actual size than the contact rate should be scaled-down simultaneously with the same ratio to get a realistic value of basic reproduction ratio. Therefore, the results of both stochastic and deterministic PSCM are exactly the same for both data sets (actual population and UTC sample). For the sake of easier comparison between the IBM simulation and PSCM analysis, we have utilized the same UTC sample data for PSCM.

B stochastic PSCM

Here, we describe how we calculate the probability that a global epidemic occurs in the Tokyo metropolitan area using a branching process [2] associated with our population size class model (PSCM). For this purpose, a global epidemic is defined as the case in which the infection never dies out in the branching process (i.e., extinction of disease did not occur). Therefore, we first calculate the probability of disease extinction for an initially infected individual living in a home population of size class n (with representative size K_n) and commuting to a work population of size class m (with representative size K_m). The probability of a global epidemic is then simply given by subtracting the extinction probability from 1.

We begin by defining the probability that an individual will commute between a given pair of home and work population size classes. As defined earlier, the number of individuals who live in a home population of size class and commute to a work population of size class is L_{nm} (Figure 2D in the main manuscript). The probability that an individual living in a home population of size class n commutes to a work population of size class m is given by $\phi^W(m|n) = L_{nm}/\sum_{m'L_{nm'}}$. In the same vein, the probability that an individual commuting to a work population of size class m lives in a home population of size class n is given by $\phi^H(n|m) = L_{nm}/\sum_{n'L_{n'm}}$.

The single primary infectious individual belongs to a commuter population of the (n_0, m_0)-th size class. Therefore, the expected numbers of secondary infections in the home and work populations are given by the basic reproductive ratio as $R^H_{n_0} = \beta K_{n_0}/\gamma$ and $R^W_{m_0} = \beta K_{m_0}/\gamma$, respectively [3, 4]. We assume complete mixing of each local population and that all local populations are initially consisted only of susceptible individuals. Susceptible hosts can be infected only by sharing either a home or a work population with an infectious host. Therefore, the number of secondarily infected hosts appearing in a non-initially infected home (work) population of size class n (m) is given by $R^W_{m_0}\phi^H(n|m_0)$ ($R^H_{n_0}\phi^W(n_0|m)$), and the

expected number of secondarily infected hosts appearing in a commuter population of the (n, m)-th size class is given by

$$R_{n_0m_0}(n, m) = \delta_{nn_0} R^H_{n_0} \phi^W(m|n_0) + \delta_{mm_0} R^W_{m_0} \phi^H(n|m_0). \quad (1)$$

Here, δ_{nm} denotes the Kronecker delta (i.e., $\delta_{nm} = 1$ if $n = m$ and $\delta_{nm} = 0$ if $n \neq m$). $R_{n_0m_0}(n, m)$ for a commuter population of the (n, m)-th size class may be non-zero only when either the home or work population is shared with commuters in the initially infected population (i.e., either $n = n_0$ or $m = m_0$). Assuming that the number of secondary infections from an infectious host follows a Poisson distribution, the probability that there will be k secondarily infected hosts in a universally susceptible commuter population of the (n, m) size class as

$$\pi_k(n_0, m_0, n, m) = \frac{\{R_{n_0m_0}(n, m)\}^k}{k!} \exp\left[-R_{n_0m_0}(n, m)\right]. \quad (2)$$

Next, we use the branching process to calculate the extinction probability of the infection. Let $Q_t(n_0, m_0)$ represent the probability that all of the infectious commuters originating from a single initially infectious individual in a commuter population of the (n_0, m_0)-th size class will go extinct within t infection cycles. This event is equivalent to the probability that all of the infectious commuters driven from k secondarily infected hosts in any possible commuter population of the (n, m)-th size class will go extinct within $t - 1$ infection cycles:

$$\begin{aligned} Q_t(n_0, m_0) &= \prod_n \prod_m \sum_{k=0}^{\infty} \{Q_{t-1}(n, m)\}^k \pi_k(n_0, m_0, n, m) \\ &= \prod_n \prod_m \sum_{k=0}^{\infty} \frac{\{Q_{t-1}(n, m) R_{n_0m_0}(n, m)\}^k}{k!} \exp\left[-R_{n_0m_0}(n, m)\right] \\ &= \prod_n \prod_m \exp\left[-R_{n_0m_0}(n, m)\left(1 - Q_{t-1}(n, m)\right)\right] \quad (3) \end{aligned}$$

Note that because this is a branching process focusing on the extinction probability of the infectious descendants of a single infected individual, subsequent infection from the secondarily infected hosts is assumed to occur mutually independently. Equation (3) can be used as a recurrence formula to calculate $Q_t(n_0, m_0)$ from $Q_0(n_0, m_0) = 0$. The last equality holds because the extinction probability within 0 generations of infection must be zero. By iterating Equation (3), we obtain $Q_t(n_0, m_0)$ for an arbitrary

value of t until it converges to a fixed-point value for $t \to \infty$. The fixed-point value can be obtained by solving the implicit relationship

$$Q_{\infty}(n_0, m_0) = \prod_n \prod_m \exp\left[-R_{n_0 m_0}(n,m)\left(1 - Q_{\infty}(n,m)\right)\right] \tag{4}$$

which gives the probability that the infection will eventually be extinct in the branching process. Because a global epidemic is defined as an exclusive event of disease extinction, the probability of a global epidemic can then be calculated as $P_G(n_0, m_0) = 1 - Q_{\infty}(n_0, m_0)$.

With reference to equation (1), above equation (4) can further be simplified as follows.

$$\begin{aligned}
Q_{\infty}(n_0, m_0) &= \prod_n \prod_m \exp\left[-R_{n_0 m_0}(n,m)\left(1 - Q_{\infty}(n,m)\right)\right] \\
&= \exp\left[-\sum_n \sum_m R_{n_0 m_0}(n,m)\left(1 - Q_{\infty}(n,m)\right)\right] \\
&= \exp\left[-\left\{R^H_{n_0} \sum_m \phi(m|n_0)\left(1 - Q_{\infty}(n_0,m)\right)\right\} - \left\{R^W_{m_0} \sum_n \phi(n|m_0)\left(1 - Q_{\infty}(n,m_0)\right)\right\}\right] \\
&= \exp\left[-\left(R^H_{n_0} + R^W_{m_0}\right) + \left\{R^H_{n_0} \sum_m \phi(m|n_0) Q_{\infty}(n_0,m)\right\} - \left\{R^W_{m_0} \sum_n \phi(n|m_0) Q_{\infty}(n,m_0)\right\}\right] \\
&= \exp\left[-\left(1 - Q_{\infty}(n,m)\right)\left(R^H_{n_0} + R^W_{m_0}\right)\right] \\
&\quad \times \exp\left[R^H_{n_0} \sum_m \phi(m|n_0)\left(Q_{\infty}(n_0,m) - Q_{\infty}(n_0,m_0)\right)\right] \\
&\quad \times \exp\left[R^W_{m_0} \sum_n \phi(m_0|n)\left(Q_{\infty}(n,m_0) - Q_{\infty}(n_0,m_0)\right)\right]
\end{aligned} \tag{5}$$

The first factor in the last equality can be interpreted as an equation to give the probability of initial extinction within the initial home and work populations (i.e., invasion of only the initial home and work populations and not the entire commute population) and the last 2 factors as a correction term for it. Neglecting this correction term allows the equation for the probability of a global epidemic to be approximated as

$$1 - P_G(n_0, m_0) = \exp\left[-P_G(n_0, m_0)\left(\frac{\beta\left(K_{n_0} + K_{m_0}\right)}{\gamma}\right)\right]. \tag{6}$$

This simply gives the probability of an epidemic in a set of initial home and work populations with a combined local population size of $K_{n_0} + K_{m_0}$.

C deterministic PSCM

(1) Epidemic dynamics among the commuter population size classes

Here, we describe our attempt to derive various properties of the epidemic dynamics of the commute network in the Tokyo metropolitan area by considering a deterministic system of difference equations for a commuter population of the (n, m)-th size class. Individuals in a commuter population of the (n, m)-th size class are classified by disease state as susceptible, infectious, or removed, allowing the total number of individuals in the (n, m) class, L_{nm}, to be decomposed as $L_{nm} = x_{nm}(t) + y_{nm}(t) + z_{nm}(t)$, where $x_{nm}(t)$, $y_{nm}(t)$, and $z_{nm}(t)$ denote, respectively, the numbers of susceptible, infectious, and recovered/removed individuals at time t. The populations within the same population size class are assumed to be statistically equivalent; that is, the epidemiological situations are assumed to be identical for all populations within the same population size class for both home and work populations. Accordingly, for an individual in the commuter population of the (n, m)-th size class, the numbers of encounters with infected individuals in their home and work populations becomes $K_n\theta_n^H(t)$ and $K_m\theta_m^W(t)$, respectively, where $\theta_n^H(t) = \sum my_{nm}(t)/\sum_m L_{nm}$ and $\theta_m^W(t) = \sum_n y_{nm}(t)/\sum_n L_{nm}$ denote the fractions of infected individuals within the home population of size K_n and work population of size K_m, respectively, at time t. For each commuter population of the (n, m)-th size class, these assumptions yield the following system of difference equations:

$$x_{nm}(t+\Delta) = x_{nm}(t)\exp\left\{-\beta K_n\theta_n^H(t)\Delta t - \beta K_m\theta_m^W(t)\Delta t\right\}, \quad (7)$$

$$y_{nm}(t+\Delta) = y_{nm}(t)\left[1 - \exp\left\{-\beta K_n\theta_n^H(t)\Delta t - \beta K_m\theta_m^W(t)\Delta t\right\}\right] - y_{nm}(t)\exp\left(-\gamma\Delta t\right), \quad (8)$$

$$z_{nm}(t+\Delta) = y_{nm}(t)\left\{1 - \exp\left(-\gamma\Delta t\right)\right\} + z_{nm}(t). \quad (9)$$

The first terms of equation (7) and equation (8) represent the infections within the home and work populations within time interval Δt. The last term of equation (8) and the first term of equation (9) represent the removal of infected individuals from the commute network (i.e., the transition from the asymptomatic state to the symptomatic state) within time interval Δt. In the actual calculations, we set $\Delta t = 1$ (i.e., the time interval is set to 1 day). For the single initially infectious individual commuting between home and work populations within the commuter population of the (n_0, m_0)-th size class, the initial conditions are represented as $x_{nm}(0) = L_{nm} - \delta_{nn_0}\delta_{mm_0}$, $y_{nm}(0) = \delta_{nn_0}\delta_{mm_0}$, and $z_{nm}(0) = 0$.

Here, we assume that the entire population initially consisted only of susceptible individuals except for the single initially infected host. Using these initial conditions, we solved equations (7)-(9) to obtain the final size of the global epidemic (the fraction of the total number of individuals who acquired the infection during the entire epidemic period), the peak time of the epidemic (the time until the total number of infected individuals attains its peak), the final size of each local epidemic, and the arrival time of the epidemic in each local population. In addition to these numerical results, we also used equations equations (7)-(9) to obtain several analytical results concerning the final size of the epidemic and the arrival time of the epidemic in each local population, as follows.

(2) Final size of the epidemic in each local population

The final size of the epidemic in each local population is defined as the fraction of individuals who have ever experience infection during the epidemic period. We denote the total numbers of susceptible, infectious, and removed individuals in the n-th home population size class as $x_n^H(t) = \sum_m x_{nm}(t)$, $y_n^H(t) = \sum_m y_{nm}(t)$ and $z_n^H(t) = \sum_m z_{nm}(t)$, and the corresponding quantities in the m-th work population class as $x_m^W(t) = \sum_n x_{nm}(t)$, $y_m^W(t) = \sum_n y_{nm}(t)$ and $z_m^W(t) = \sum_n z_{nm}(t)$, respectively. The final sizes of the local epidemic in the n-th home population size class Ψ_n^H and the m-th work population size class Ψ_m^W are defined as $\Psi_n^H \equiv z_n^H(\infty)/L_n^H$ and $\Psi_m^W \equiv z_m^W(\infty)/L_m^W$, respectively. To calculate these values, we further define the final size of the epidemic within the commuter population of the (n,m)-th size class as

$$\Psi_{nm} \equiv \frac{z_{nm}(\infty)}{L_{nm}} = 1 - \frac{x_{nm}(\infty)}{L_{nm}}. \tag{10}$$

Equation (7) and equation (9) are then combined to obtain

$$\begin{aligned} x_{nm}(t) &= x_{nm}(0)\exp\left[-\beta\Delta t\frac{K_n}{L_n^H}\sum_{m'}\sum_{i=1}^{T} y_{nm'}(t-i\Delta t)\right]\exp\left[-\beta\Delta t\frac{K_m}{L_m^W}\sum_{n'}\sum_{i=1}^{T} y_{n'm}(t-i\Delta t)\right] &(11)\\ y_{nm}(t) &= \frac{1}{1-e^{-\gamma\Delta t}}\left(z_{nm}(t+\Delta t)-z_{nm}(t)\right) &(12)\end{aligned}$$

Where $t = T\Delta t$, we substituted equation (12) in equation (11) with $t = \infty$ and using $x_{nm}(0) \cong L_{nm}$ we have

$$x_{nm}(\infty) = L_{nm}\exp\left[-\frac{\beta\Delta t}{1-e^{-\gamma\Delta t}}\left(\frac{K_n}{L_n^H}\sum_{m'} z_{nm'}(\infty) + \frac{K_m}{L_m^W}\sum_{n'} z_{n'm}(\infty)\right)\right] \tag{13}$$

and combining this with equation (10), we have a set of equations to determine Ψ_{nm}:

$$\Psi_{nm} = 1 - \exp\left[-\frac{\beta\Delta t}{1-e^{-\gamma\Delta t}}\left(\frac{K_n}{L_n^H}\sum_{m'} L_{nm'}\Psi_{nm'} + \frac{K_m}{L_m^W}\sum_{n'} L_{n'm}\Psi_{n'm}\right)\right] \tag{14}$$

This system of equations can be solved numerically by recursively inserting Ψ_{nm} from the right side as Ψ_{nm} on the left side, starting from $\Psi_{nm} = 1$, until the result converges to a fixed point. Needless to say, the above values also give the final size of the epidemic in each local population within the specified population size class. Moreover, the final size of the global epidemic can also be calculated from Ψ_{nm} as

$$\Psi = \frac{\sum_n \sum_m z_{nm}(\infty)}{N} = \frac{\sum_n \sum_m L_{nm}\Psi_{nm}}{N} \tag{15}$$

(3) Arrival time of the epidemic in each local population

The arrival time of the epidemic in each local population is defined as the time until the infected individual first appears in the local population since the epidemic has started. This can be calculated from the time course of the number of infected individuals (i.e., $y_n^H(t)$ and $y_m^W(t)$) in each population size class as follows. To define the time t_n^H at which the first infected individual appears in a home population of size class n, we first note that $y_n^H(t)$ is for the total number of infected individuals in such a size class. As the total number of hosts in the home population size class n is L_n^H, and as the representative population size of class n is K_n, there are L_n^H/K_n such populations. Therefore, the number of infected hosts at time t in each local home population of size class n is given by $y_n^H/(L_n^H/K_n)$, and t_n^H and, similarly, t_m^W, are defined as

$$\begin{aligned} \frac{y_n^H(t_n^H)}{L_n^H/K_n} &= 1 \\ \frac{y_m^W(t_m^W)}{L_m^W/K_m} &= 1 \end{aligned} \tag{16}$$

To obtain the approximate formula (including the logarithmic dependence mentioned in the main text) for the arrival times we linearize the system of equations (7)-(9) as

$$\begin{aligned} y_{nm}(t+\Delta t) &= L_{nm}\left\{\beta K_n \frac{\sum_m y_{nm}(t)}{L_n^H}\Delta t + \beta K_m \frac{\sum_n y_{nm}(t)}{L_m^W}\Delta t\right\} - \exp(-\gamma\Delta t) y_{nm}(t) \\ &= \sum_i \sum_j \left\{\beta K_n \frac{L_{nm}}{L_n^H}\Delta t \delta_{in} + \beta K_m \frac{L_{nm}}{L_m^W}\Delta t \delta_{jm} + e^{-\gamma \Delta t}\delta_{in}\delta_{jm}\right\} y_{ij}(t) \qquad (17) \end{aligned}$$

By denoting the total number of population size classes as M_H for the home populations and M_W for the work populations, we then interpreted $y_{nm}(t)$ as a vector with $M_H M_W$ elements with 2 indices n ($n = 1, 2, \cdots, M_H$) and m ($m = 1, 2, \cdots, M_W$). The coefficients within the curly bracket of equation (17) could then be read as an $M_H M_W \times M_H M_W$ matrix. This matrix has $M_H M_W$ eigenvalues and corresponding eigenvectors (i.e., left and right eigenvectors forming a biorthogonal set). As both the left and right eigenvectors spanned a complete set in $M_H M_W$ space, the solution of the linearized equation (17) under the initial condition $y_{nm}(0) = \delta_{nn_0}\delta_{mm_0}$ can be expanded using the right eigenvectors as follows.

$$y_{nm}(t) = \sum_{n'} \sum_{m'} c_{n'm'} v_{nm}^{(n'm')} \left(\rho_{n'm'}\right)^t \qquad (18)$$

where the (n', m')-th eigenvalue is denoted as $\rho_{n'm'}$ and the (n, m)-th element of the corresponding right eigenvector as $v_{nm}^{(n'm')}$. Because the right eigenvector and the left eigenvector form a biorthogonal set, the expansion coefficient $c_{n'm'}$ is given from the inner product between the (n', m')-th left eigenvector and the initial vector $y_{nm}(0)$ as

$$c_{n',m'} = \sum_n \sum_m u_{nm}^{(n'm')} y_{nm}(0) = u_{n_0 m_0}^{(n'm')}. \qquad (19)$$

Here, the (n, m)-th element of the (n', m')-th right eigenvector is denoted by $u_{nm}^{(n',m')}$ and the initial condition in which the initial infected individual is only in a commuter population of the $(n_0 m_0)$-th size class is used. Up to now, equation (18) and equation (19) give the formal solution for the linearized system of equation (17). As a second approximation step, we will assume exponential growth of the infected populations at a rate given by the largest real eigenvalue ρ. The left and right eigenvectors corresponding to the largest real eigenvalues are denoted as u_{nm} and v_{nm}, respectively. Because the linearized coefficient matrix is a non-negative matrix (i.e., the Perron-Frobenius theorem is applicable), the dominant eigenvalue is purely real; moreover, the elements of the corresponding left and right eigenvectors are also

purely real. This means that at the long time limit, the contribution from the eigenstate with eigenvalue ρ would exceed the other eigenstates in the expansion of equation (18). Under this exponential growth approximation, the elements of the corresponding right eigenvector gives the relative ratio between the populations in the exponential growth phase and the elements of the corresponding left eigenvector gives the greproductive value,h which represents the contribution from each population to the exponential growth. Using this exponential growth approximation, it is possible to approximate the expansion of equation (18) as

$$y_{nm}(t) \cong u_{n_0 m_0} v_{nm} (\rho)^t . \tag{20}$$

From this, we calculate y_n^H and y_m^W and then insert the results into the definition of the arrival time of the epidemic in equation (16) to obtain the arrival times of the epidemic as follows

$$\begin{aligned} t_n^H &= \frac{1}{\ln \rho} \left\{ \ln \frac{L_n^H}{v_n^H} - \ln u_{n_0 m_0} - \ln K_n \right\} \\ t_m^W &= \frac{1}{\ln \rho} \left\{ \ln \frac{L_m^W}{v_m^W} - \ln u_{n_0 m_0} - \ln K_m \right\} \end{aligned} \tag{21}$$

where $v_n^H = \sum_m v_{nm}$ and $v_m^W = \sum_n v_{nm}$.

The logarithmic population size dependence appears in the third factor of equation (21) (i.e., $\ln K_n$ and $\ln K_m$). However, the first factor of equation (21) contains a population size class dependence. In addition, both L_n^H (L_m^W) and v_n^H (v_m^W) should also have depend on the population size class. However, the calculated numerical results show that the population size class dependence of the first factor is relatively small (i.e., the dependence is somehow cancelled out in the ratio L_n^H/v_n^H (L_m^W/v_m^W)) relative to that of the third factor. Therefore, the clear logarithmic population size dependence in the arrival time of the epidemic in each local population that we observed in the IBM must have originated from the third factor of equation (21). Although the actual calculation of equation (21) requires a numerical eigenvalue calculation, it gives the formal explicit solution, which is very effective for examining the epidemic parameter dependence.

References

1. (2007) The 10th Urban Transportation Census Report (in Japanese). Technical report, Ministry of Land Infrastructure Transport Tourism.

2. Feller W (2008) An Introduction to probability theory and its applications (Vol.1). Jon Wiley & Sons.

3. Anderson RM, May RM (1992) Infectious Diseases of Humans: Dynamics and Control (Oxford Science Publications). Oxford: Oxford university press.

4. Murray JD (2002) Mathematical Biology: I. An Introduction (Interdisciplinary Applied Mathematics). Springer.
